# *KRAS*: Druggable at Last

**DOI:** 10.1093/oncolo/oyad014

**Published:** 2023-03-18

**Authors:** Benjamin O Herzberg, Gulam A Manji

**Affiliations:** Department of Medicine, Division of Hematology/Oncology, Columbia University Irving Medical Center, New York, NY, USA; Department of Medicine, Division of Hematology/Oncology, Columbia University Irving Medical Center, New York, NY, USA

## Abstract

The approval of adagrasib for non–small cell lung cancer is a milestone in drug development. This commentary highlights the history of the research that led to this breakthrough that will help many patients with KRAS-mutated cancers to live longer and better.

In early December, the US Food and Drug Administration granted accelerated approval to adagrasib, a mutant-selective inhibitor of the Kirsten rat sarcoma viral homolog (*KRAS*), for the treatment of metastatic non-small cell lung cancer (NSCLC) harboring a *KRAS* G12C mutation after one prior therapy.^1^ Adagrasib now joins sotorasib,^2^ which received accelerated approval in May, 2021 for the same indication. In some ways, the arrival of adagrasib simply represents a continuation of what we learned a year prior: that *KRAS* was, at long last, an oncogene that we could shift from the “undruggable” column to the “druggable” one.^3^ But this approval underlies something else: a confluence of new biology, new chemistry, new clinical questions, and new avenues of drug discovery. From this vantage point, the approval of adagrasib looks not so much like the beginning of the end for *KRAS*-mutated cancers, but something much closer to the end of the beginning.

*KRAS* was among the first discovered human oncogenes. It was a member of the initial group of so-called viral oncogenes whose existence was traced not to the retroviruses in which they were initially identified but instead to the endogenous genome of the cell—proving that the keys to cancer were not outside, but within, the human genome.^4^ Over time, *KRAS* grew in importance, chiefly for 2 features: it is among the most mutated of all oncogenes across all histologies and its biology and biochemistry made it pharmacologically untouchable.^5^ The KRAS protein is a GTPase that integrates activity from upstream receptor tyrosine kinases (RTKs) and signals downstream to regulate cell growth. To do this, it cycles between a protein “on” state, or KRAS(on), in which it is bound to GTP, and a KRAS(off) state, in which it is GDP-bound. KRAS contains an internal system to shut itself off, by activating its GTPase function; a variety of helper proteins regulate KRAS activity by shifting the balance between KRAS(GDP) and KRAS(GTP). Oncogenic mutations in KRAS shift the balance toward perpetual activity—and unchecked growth. It is an elegant system but so elegant that almost no exogenous molecule could directly disrupt it. In the 2000s, an era where oncogenic kinase after kinase fell to drug hunters and medicinal chemists, KRAS played on, unperturbed. It has a picomolar affinity for GTP (which is micromolar abundant in cells), an interaction nothing could displace. It had no obvious allosteric sites. It was uncrackable safe.

The insight that would eventually become sotorasib and adagrasib took advantage of the rise—or rerise—of covalent molecules as human drugs.^6^ The (re)discovery of the uses of covalency has allowed for overcoming resistance and developing more selective cancer drugs in other settings, such as Bruton’s tyrosine kinase (BTK) inhibitors or the ­3rd-generation epidermal growth factor receptor (EGFR) TKI, osimertinib. But in the case of targeting KRAS, it was covalency itself that was the starting point. The insight was first reported by Ostrem and Shokat,^7^ who noted that one particular KRAS mutation, the p.G12C mutation, replaced an inert glycine with a reactive cysteine. They screened small cysteine-reactive molecular fragments against it. Building up from these fragments, they discovered a cryptic pocket adjacent to the GDP binding site. They synthesized a molecule that could bind the KRAS(GDP), locking it in that state, such that it was unable to cycle to GTP-bound KRAS(on). GTP-GDP shuttling, in the presence of an inhibitor, turns the system off.^8^ With this foothold, more selective molecules followed. From tool compound to efficacy in the clinic took a mere 8 years. Add one more, and now we have our 2nd KRAS G12C-allele specific, GDP-state inhibitor: adagrasib.

A key question is: are adagrasib and sotorasib different? In many ways not. They are both allele-specific inhibitors and covalent drugs. With the caveat that single-arm studies should not be directly compared even more so than studies with comparator arms should, response rates and side effect profiles look broadly similar. In NSCLC, the response rate is about 35%-45%; the median response time is about 8 months. They both have their highest reported efficacy in NSCLC, with monotherapy response rates in PDAC and CRC with G12C mutations probably about a third to half the NSCLC rate, with some variation.^9-11^

But there are some differences—some driven by the luck and pace of clinical development, and some which may be because of the differences in the drugs themselves. A reasonable reading of the 2 pivotal, single-arm trials would be that adagrasib trades a slightly higher response rate than sotorasib for a slightly higher rate of primary toxicity, mostly diarrhea, nausea, fatigue, and LFT elevations.^12^ Discontinuation, dose holds, and dose reductions of adagrasib were reported at a slightly higher rate than for sotorasib. Adagrasib, importantly, has more data for use with brain metastases than does sotorasib; preclinical studies indicate adagrasib penetrates the cerebrospinal fluid,^13^ and the reported response rate in the central nervous system is 33%. Although most of these patients had prior radiation therapy for brain metastases, the early data indicate that responses in the brain are common and benefits appeared associated with responses in the body. In a retrospective review of the CodeBreak 100 data focused on brain metastases,^14^ sotorasib had a high disease control rate in stable, radiotherapy-treated disease, some hint of responses, and is currently enrolling prospective cohorts evaluating its activity in untreated disease (NCT04185883). Sotorasib is more advanced in development, and therefore has randomized data versus SOC, recently displaying longer PFS, and better tolerability, when compared ­head-to-head with docetaxel, a reasonably standard 2nd-line therapy for NSCLC (though without ramucirumab).^15^ But there was no overall survival benefit observed—a surprise, perhaps, though the trial was not powered for this endpoint, and crossover was common—appropriately, given data on drug activity. Adagrasib is in the ring with docetaxel now (NCT04685135).

These drugs also have another trait in common: by the standards of TKIs used for other single oncogene-driven lung cancers, observed response rates are lower and median durations of response shorter. This has several important, interrelated implications. First, they have accelerated approval initially as 2nd-line therapies, not first-line. Second, resistance mechanisms appear much more varied, so there is no obvious next strategy when patients fail, and they fail more quickly.^16,17^ Finally, and as a result, a huge interest has developed in combining both of these drugs with others—chemotherapy, immune checkpoint blockade, and other adjunctive RAS-targeted therapies. If successful we will be slicing pockets of NSCLC ever more finely: KRAS G12C mutated, PD-L1 high: G12C inhibitor/checkpoint inhibitor; KRAS G12C mutated, PD-L1 zero: G12C inhibitor/chemotherapy; KRAS G12C mutated, STK11/KEAP1 mutation: G12C inhibitor/novel strategy^18^; and so on. But despite promising preclinical models of combination therapies, success here is not assured. Pembrolizumab or atezolizumab combined with sotorasib led to marked rates of hepatic enzyme elevation in a trial partially reported this summer;^19^ development is ongoing with new strategies, such as a lead-in of sotorasib followed by ICI, rather than concurrent initiation. Mirati has shown some data for adagrasib-ICI combination as an early press release, with less reported toxicity, but a very minimal duration of follow-up. It may be here that differences in the pharmacology and pharmacokinetics of each of these drugs will most play out. Whether we will find “synergy,” or merely 2 cross-tolerable therapies that complement (or impair) one another, remains to be seen.

Similarly, for the minority of CRC and PDAC patients with G12C mutations—which may confer a poorer prognosis^20,21^—relatively lower monotherapy response rates have accelerated combination strategies to the forefront.^9-11^ In a recent report, a combination of adagrasib with cetuximab, an EGFR-blocking monoclonal antibody, led to a markedly higher response rate in colorectal cancer with *KRAS* G12C compared to monotherapy or historical cetuximab data.^9^ Prior work has reported that upon treatment with an inhibitor, KRAS dependence may be bypassed by cells upregulating other MAP kinase (MAPK) pathway inputs. This has suggested that dual-targeting of the pathway, whether by targeting an upstream RTK or other MAPK signalers such as SHP-2, may be required for durable responses.^22,23^ Resistance patterns to adagrasib/cetuximab combination therapy also appear different than resistance to monotherapy, at least for colorectal cancers, and lead to unique biology when KRAS inhibitors are withdrawn.^24^ These early data underscore the lesson that even common mutations can lead to very different biology in different histologies. With direct KRAS inhibitors, we have tools to rapidly innovate such strategies in near real-time to benefit patients.

And if that was not enough, there are more than a ­half-dozen new G12C-specific compounds in development (Fig. 1).^25^ Some are structurally and mechanistically similar. Some have similar activity but are chemically unique. Most target the KRAS (GDP) state, like sotorasib and adagrasib, by binding to the known “switch-II” pocket and preventing cycling to KRAS (GTP). But others, like RMC-6291, target the KRAS(on) state, by sterically occluding effector proteins from binding to active KRAS.^26^ Will any improve response rates? Durations? Will they have different toxicities, or the same? Will combination therapies be different—and more or less important? Will our clinical development programs be able to effectively allocate patients to trials and identify the best way forward in a highly crowded field full of single-arm studies? We will find out in the coming years.

**Figure 1.**
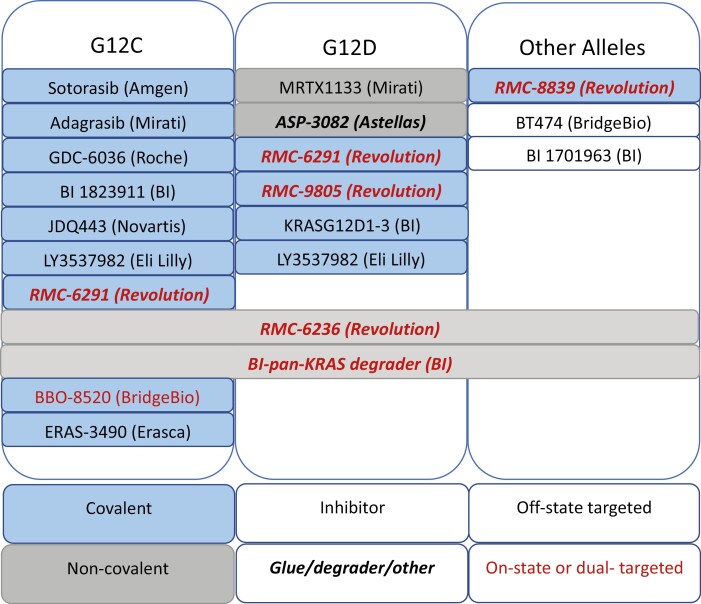
Disclosed *KRAS* inhibitors and their properties. Many direct KRAS-targeted molecules are in current clinical development. These can be divided by their specificity (single allele versus pan-RAS); mode of chemical binding (covalent versus non-covalent); mechanism of action (inhibitor versus degrader or glue); and whether they target a single or both states of the protein.

The G12C mutation may be the most common in NSCLC—but it is a minority of KRAS alleles across all other cancers. In gastrointestinal cancers, G12D and G12V predominate. In pancreas cancer alone, 1 of these 2 is found in 65% of tumors.^5^ There are other oncogenic mutations in G13 and Q61 with less well-understood biochemical implications. These mutations have different properties—starting with different intrinsic hydrolysis rates for GTP^27^—and, perhaps more importantly, no highly-reactive substitution from which to build a covalent drug. It is still unknown whether all KRAS alleles will be equally targetable with better chemistry—or whether we will run into intrinsic limitations imposed by the biochemical properties of the alleles themselves.^28^ It is worth remembering how many past clinical strategies to indirectly target KRAS-mutant tumors have had marginal, at best, impact in the clinic.

But the fishing expedition for G12C inhibitors which started with a tiny cysteine-reactive fragment has sparked a broader, analogous one, whereby the structural knowledge from adagrasib and sotorasib has opened up new avenues to directly attack different KRAS alleles (Fig. 1).^5^ Adagrasib was the starting point for one of these campaigns, with many rounds of chemical optimization used to generate a specific, but non-covalent, G12D inhibitor, MRTX1133.^29^ An early tool compound to attack G12S, by acetylating the acquired serine, has been published.^30^ With the door opened, there is now a proliferating cornucopia of directly targeting KRAS strategies, from such non-covalent G12D inhibitors, to ­allele-specific G12D PROTACs (ASP-3082, Astellas, in clinic), to pan-RAS PROTACs (Boehringer Ingelheim, disclosed), to unique pan-RAS glue-like compounds such as RMC-6236 (Revolution Medicines, in clinic), or RAS:PI3K interrupters (BridgeBio, disclosed). Although most of these compounds are in early dose escalation studies, the trials are likely to enroll quickly, given the prevalence of non-G12C mutations, the established proof-of-principle with adagrasib and sotorasib, and the lack of other compelling options for these patients. For oncologists treating both lung and non-lung cancers, and for patients, this is a welcome explosion—and one that we hope, in hindsight, will have brought major improvements in therapy.

The approval of adagrasib is therefore a milestone in drug development, and oncology. Many patients with KRAS G12C mutated cancers will likely live longer, and better, because of it and its companion-competitor drug sotorasib. They are mechanistically novel compounds sitting on the shoulders of nearly 50 years of basic oncology and chemistry research. But they are also not, nearly, the last word. They are much more like the first.
